# Role of cytotoxic T cells and PD-1 immune checkpoint pathway in papillary thyroid carcinoma

**DOI:** 10.3389/fendo.2022.931647

**Published:** 2022-11-28

**Authors:** Sohini Banerjee, Uma Nahar, Divya Dahiya, Soham Mukherjee, Pranab Dey, Rijuneeta Gupta, Bishan Radotra, Naresh Sachdeva, Ashwani Sood, Sanjay Kumar Bhadada, Anil Bhansali

**Affiliations:** ^1^ Department of Histopathology, Postgraduate Institute of Medical Education and Research, Chandigarh, India; ^2^ Department of General Surgery, Postgraduate Institute of Medical Education and Research, Chandigarh, India; ^3^ Department of Endocrinology, Postgraduate Institute of Medical Education and Research, Chandigarh, India; ^4^ Department of Cytology and Gynaecological Pathology, Postgraduate Institute of Medical Education and Research, Chandigarh, India; ^5^ Department of Otolaryngology (ENT), Postgraduate Institute of Medical Education and Research, Chandigarh, India; ^6^ Department of Nuclear Medicine, Postgraduate Institute of Medical Education and Research, Chandigarh, India

**Keywords:** papillary thyroid carcinoma, tumor microenvironment, programmed death-1, CD8 T cell, anti tumor immunity

## Abstract

**Background:**

Lymphocytic thyroiditis (LT) is frequently seen in the tumor microenvironment (TME) of papillary thyroid carcinomas (PTCs). However, the characteristic of these tumor-infiltrating lymphocytes (TILs) is not well understood.

**Objective:**

We aim to define the TME of PTC cases by characterizing the TILs.

**Design:**

This is a cross-sectional observational study.

**Patients:**

We enrolled 29 PTC (23 having concurrent LT), 14 LT, and 13 hyperplastic nodules with LT (HN) patients from January 2016 to December 2020.

**Measurements:**

Immunohistochemical (IHC) expression of CD8, FoxP3, PD-1, and PD-L1 was studied in PTC with LT and compared with HN. PD-1 and PD-L1 expression was correlated at the mRNA level by quantitative real-time PCR. Immunophenotyping of TILs was done in FNAC samples of PTC and LT by flow cytometry.

**Results:**

IHC revealed the presence of CD8^+^ cytotoxic T lymphocytes (CTLs) and FoxP3^+^ T regulatory cells (Tregs) in 83% and 52% of PTC with LT cases, respectively. Flow cytometric analysis of the PTC samples revealed a significant abundance of CTL compared with Treg and a higher CTL with lower Treg counts compared with LT. On IHC, PD-1 positivity was noted in 56.5% of PTC with LT cases, while intermediate PD-L1 positivity was found in 70% of the cases. There was a significant upregulation of PD-1 mRNA in PTC with LT. A significant correlation was noted with PD-L1 expression with lymph node metastasis and presence of Treg cells.

**Conclusions:**

Increased expression of PD-1 and PD-L1 in the TME of PTC may provide a potential molecular mechanism for tumor survival despite the predominance of CTLs, possibly through their inactivation or exhaustion.

## Introduction

Papillary thyroid carcinoma (PTC) is the predominant histological subtype of thyroid carcinomas accounting for 90% of thyroid cancers. Nearly 10% of patients with PTC develop recurrence or metastasis, and the rate is higher in aggressive histopathological variants including the tall cell and hobnail variant (1). The pathophysiological mechanisms including tumor-mediated immune suppression underlying aggressive variants of PTC need to be explored. It is well-established that host immune cells can recognize and eliminate malignant cells. However, the precise mechanism leading to immune escape of tumor cells, including downregulation of antigen recognition, expression of immune-inhibitory ligands, and recruitment of suppressor immune cell populations, is yet to be determined (2).

The tumoral expression of immunosuppressive molecules and the specific patterns of tumor-infiltrating lymphocytes (TILs) have been shown to predict cancer recurrence and survival in solid malignancies (3, 4). The evolution of immune checkpoint inhibitors as anticancer treatment options represents one of the most successful approaches in cancer drug discovery for the past few years (5). In 2011, the first immune checkpoint inhibitor (ipilimumab as an anti-CTLA-4 antibody) was approved by the US FDA for the treatment of melanoma and created a footstep in cancer immunotherapy (6, 7). Currently, two classes of immunotherapy which have been approved by the FDA for clinical use include inhibitors of programmed death receptor 1 (PD-1) or its ligand (PD-L1) and cytotoxic T-cell lymphocyte-associated protein 4 (CTLA-4) (8). By targeting PD-1 and PD-L1 interaction, the immune checkpoint inhibitors can reactivate cytotoxic T cells to act against cancer cells. PD-1 is a key immune checkpoint receptor mainly expressed by activated T cells which interacts with its ligand PD-L1, expressed on tumor cells. Through this interaction in the tumor microenvironment (TME), cancers mediate immunosuppression and escape immune elimination. The inhibition of the interaction between PD-1 and PD-L1 can enhance T-cell responses *in vitro*, which may result in antitumor effect. Additionally, patients with PD-L1+ tumors have better response to anti-PD-1 treatment than those with PD-L1-negative tumors (9–11).

Tumor-associated lymphocytic thyroiditis (LT) is frequently observed in the TME of PTCs. In Hashimoto’s thyroiditis (HT), lymphocytic infiltration leads to thyroid tissue destruction with consequent hypothyroidism, while in PTC with LT, it is usually not associated with the development of concurrent hypothyroidism. Hence, it may suggest an interplay between the TILs and tumor resulting in inactivation of these immune cells. However, data to define the TME based on the characteristic and role of TILs in PTC are sparse in the literature. Bagnasco et al. (12) reported a higher proportion of cytotoxic T cells with natural killer (NK) or lymphokine-activated killer activity in PTC with lymphocytic thyroiditis (LT) compared with those occurring in autoimmune thyroid disorders, e.g., HT. In the present study, we aimed to define the TME of PTC cases by characterizing the TILs and compared them with benign thyroid disease and also analyzed the expression pattern of PD-1 and PD-L1 in the TME.

## Materials and methods

The study was conducted at the Postgraduate Institute of Medical Education and Research (PGIMER), Chandigarh from January 2015 to December 2020. Ethical clearance was obtained from the PGIMER Institute Ethics Committee. On the one hand, histopathologically proven classical PTC with LT and hyperplastic nodules with LT cases were included for immunohistochemical evaluation. Fresh tumor tissues from all these patients were collected at the time of surgery and transferred to RNAlater for subsequent RNA extraction. On the other hand, thyroid fine-needle aspiration cytology (FNAC) samples from consecutive cases of PTC and LT were collected for flow cytometric analysis (FCM). Cases and controls of both sexes with age above 18 years were included in the study.

Informed consent from all the patients was collected. The demographic and clinical details of PTC patients including age, sex, tumor stage, lymph node metastasis, extrathyroidal extension (ETE), and distant metastasis were retrieved from clinical records and histopathology reports from the Departments of Endocrinology, Nuclear Medicine, and Histopathology, respectively. The histologic subtypes was confirmed by reviewing the hematoxylin and eosin (H&E)- stained slides by two observers (UN and SB) without bias.

### Immunohistochemistry

The formalin-fixed, paraffin-embedded (FFPE) tissue blocks were used for immunohistochemistry (IHC) using standard protocol (13). The primary antibody to human PD-1 (clone EH33; mouse mAb, Cell Signaling Technology: Massachusetts, USA) and PD-L1 (clone E1L3N, rabbit mAb, Cell Signaling Technology: Massachusetts, USA) at room temperature was added and incubated for 45–60 min. The slides were washed with 1× PBST and secondary antibody was added to the slides followed by DAB and hematoxylin counterstaining.

### Immunohistochemical scoring

The immunoreactivity of CD8, FoxP3, and PD-1 was scored semiquantitatively as described by Zha et al. (14). The intensity of staining was graded as 0 (negative), 1 (weak), 2 (moderate), and 3 (strong). The frequency was graded from 0 to 4 by the percentage of positive cells as follows:

- Grade 0, <3%
- Grade 1, 3%–25%
- Grade 2, 25%–50%
- Grade 3, 50%–75%
- Grade 4, >75%

The index score was the product of multiplication of the intensity and frequency grades, which was then graded into a 4-point scale:

- Index score 0 (−), product of 0
- Index score 1 (+), products of 1 and 2
- Index score 2 (++), products of 3 and 4
- Index score 3 (+++), products of 6–12

The immunoreactivity of PD-L1 was followed by the tumor proportion score (TPS) and combined positive score (CPS) approaches.

TPS scoring was done by the following method:

(No. of PD-L1 stained tumor cells/No. of tumor cells) × 100

CPS scoring was done by the following method:

(No. of PD-L1 stained tumor and inflammatory cells/No. of tumor cells) × 100 (15).

### Classification of TME

The tumor microenvironment was classified based on PD-L1 and PD-1 expression as type 1 (PD-L1+/PD-1+), type 2 (PD-L1−/PD-1−), type 3 (PD-L1+/PD-1−), and type 4 (PD-L1−/PD-1+) (16).

### RNA extraction and cDNA synthesis

Total RNA was extracted from fresh tumor tissues of PTC and hyperplastic nodule cases collected at the time of surgery using the mirVana PARIS Kit according to the manufacturer’s instructions (Invitrogen: Massachusetts, USA) and quantified spectrophotometrically. The RNA integrity was verified by 1% agarose gel electrophoresis. The RNA was reverse-transcribed using 1 μg of total RNA in a total volume of 20 μl using cDNA synthesis kit (Bio-Rad: California, USA). The cDNA prepared was stored at −20°C for quantitative RT-PCR.

### Quantitative real-time RT-PCR

The expression of PD-1 and PD-L1 mRNA was determined by quantitative real-time (RT) PCR using the LightCycler system (Applied Biosystems: Massachusetts, USA) according to the protocol used by Feilchenfeldt et al. (17).

### Flow cytometry

#### Lymphocyte isolation and staining

Thyroid FNA samples were collected in a 3-ml EDTA tube containing 1 ml of PBS. Density gradient centrifugation at 1,800 rpm for 30 min was done by Ficoll solution. Buffy coat containing lymphocytes was isolated followed by surface staining in the dark by adding 4 μl of fluorochrome-conjugated antibodies against human CD45, CD3, CD4, CD8, CD20, and CD25 which was incubated for 20 min. The supernatant was discarded and a subset of cells was permeabilized with Cytofix/Cytosperm fixation and permeabilization solution and proceeded for the intracellular staining of FoxP3 transcription factor and IL-17A cytokine (18). Data were acquired using a BD FACSCanto flow cytometer.

#### Flow cytometry data analysis

Analysis of the cell population was performed based on a four-step criteria. The first step was the gating of lymphocytes on the basis of forward and side scatter characteristics. The second step was the removal of dead cells, debris, and doublets. The third step was the gating of T cells and non-T cells on the basis of CD3 expression. From the CD3-gated cells, CD4 *vs*. CD8-positive cells were plotted which will allow the identification of discrete CD4 and CD8 cell populations. Subsequent analyses were done on these individual cell populations.

### Statistical analysis

Data analysis was performed using SPSS 23.0 and GraphPad Prism 7.0 statistical software. Categorical data were evaluated using the chi-squared test or Fisher’s exact probability test as appropriate. The differences were considered significant when the *P*-value was <0.05.

## Results

A total of 29 PTC patients were enrolled in the study, of which IHC was done in 23 patients (group 1, female:male ratio 6.7:1) and FCM was done in 7 patients (group 2, female:male ratio 1.3:1). One patient was common in both groups. The clinical details are depicted in **Table 1**. The overall survival in group 1 was 94% (16/17) after a mean follow-up of 3.2 ± 0.52 years and 100% (3/3) in group 2 after a mean follow-up of 2.5 ± 0.34 years. Four out of those who survived (*n* = 20) had shown disease progression during the follow-up period.

**Table 1:** Clinical details of patients.

Parameters	Group 1 (*n* = 23)	Group 2 (*n* = 7)
Mean age	37.52 ± 13.59^a^	44 ± 14.34^a^
Mean tumor diameter (cm)	2.6 ± 0.35^a^	3.8 ± 1.2^a^
Histological subtype	Classical 91% (21/23) Follicular variant 9% (2/23)	Classical 100% (7/7)
Tumor diameter		
≤2 cm >2 to ≤4 cm >4 cm	47% (8/17) 41% (7/17) 12% (2/17)	33% (2/6) 50% (3/6) 17% (1/6)
Cervical LNM	72% (16/22)	33% (2/6)
Mean follow-up (years)	3.2 ± 0.52^a^	2.5 ± 0.34^a^
Distant metastasis	17.6% (3/17)	33% (1/3)
Mortality	6% (1/17)	0 (0/3)

a Data presented as mean ± SEM.

### Immunophenotyping of TILs in FFPE tissue by IHC

**Figure 1:**
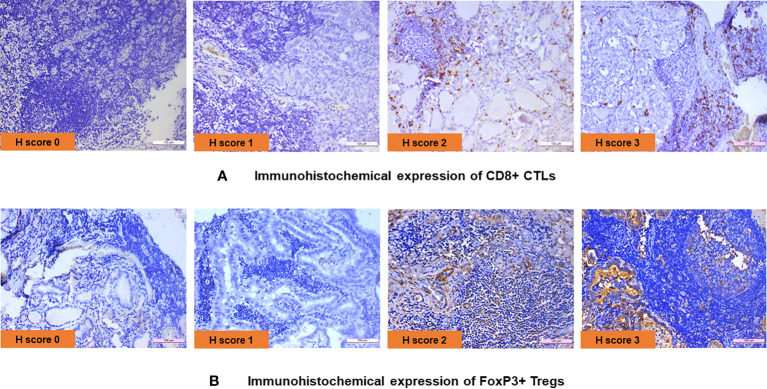
**(A)** Immunohistochemical expression of CD8^+^ CTLs. **(B)** Immunohistochemical expression of FoxP3^+^ Tregs.

**Figure 2:**
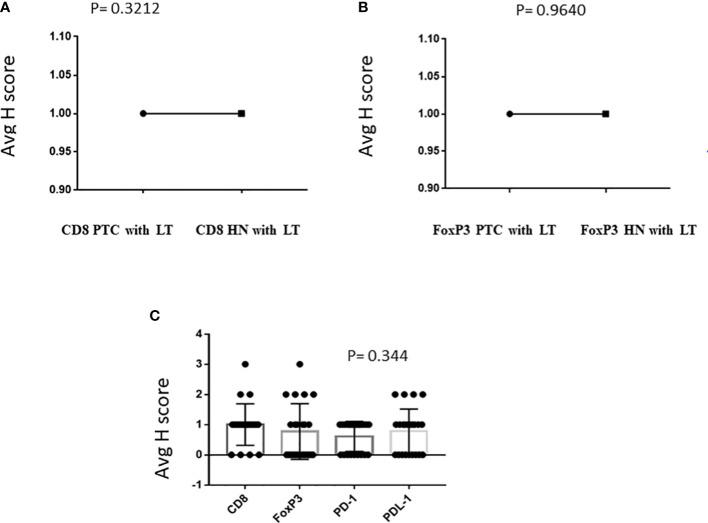
**(A–C)** Relative expression of CD8 and FoxP3 in PTC with LT.

IHC was performed in 23 PTC with LT cases and 13 cases of hyperplastic nodules with LT. In all cases of PTC with LT, adjacent thyroid follicles showed evidence of lymphocytic infiltration with lymphoid follicle formation. The IHC revealed the presence of CD8^+^ CTL in 19/23 (83%) PTC with LT cases with an intensity varying from 1+ to 3+ (**Figure 1A**), whereas FoxP3^+^ Treg cells were present in 12/23 (52%) cases with an intensity of 1+ to 3+ (**Figure 1B**). We also observed a similar expression pattern of CD8^+^ CTLs and Foxp3^+^ Tregs in hyperplastic nodules with LT cases (11/13 = 85% and 8/15 = 53%, respectively). **Figures 2A, B** show that the average *H* score of CD8^+^ CTLs and Foxp3^+^ Tregs was similar in PTC with LT cases and hyperplastic nodules with LT cases (*P* = 0.3212 and 0.9640, respectively). Also, there was a statistically insignificant relative abundance of CD8^+^ CTLs compared with FoxP3^+^ Treg cells in PTC with LT (*P* = 0.3440, **Figure 2C**).

### Immunohistochemical expression of PD-1 and PD-L1 in PTC with LT

**Figure 3:**
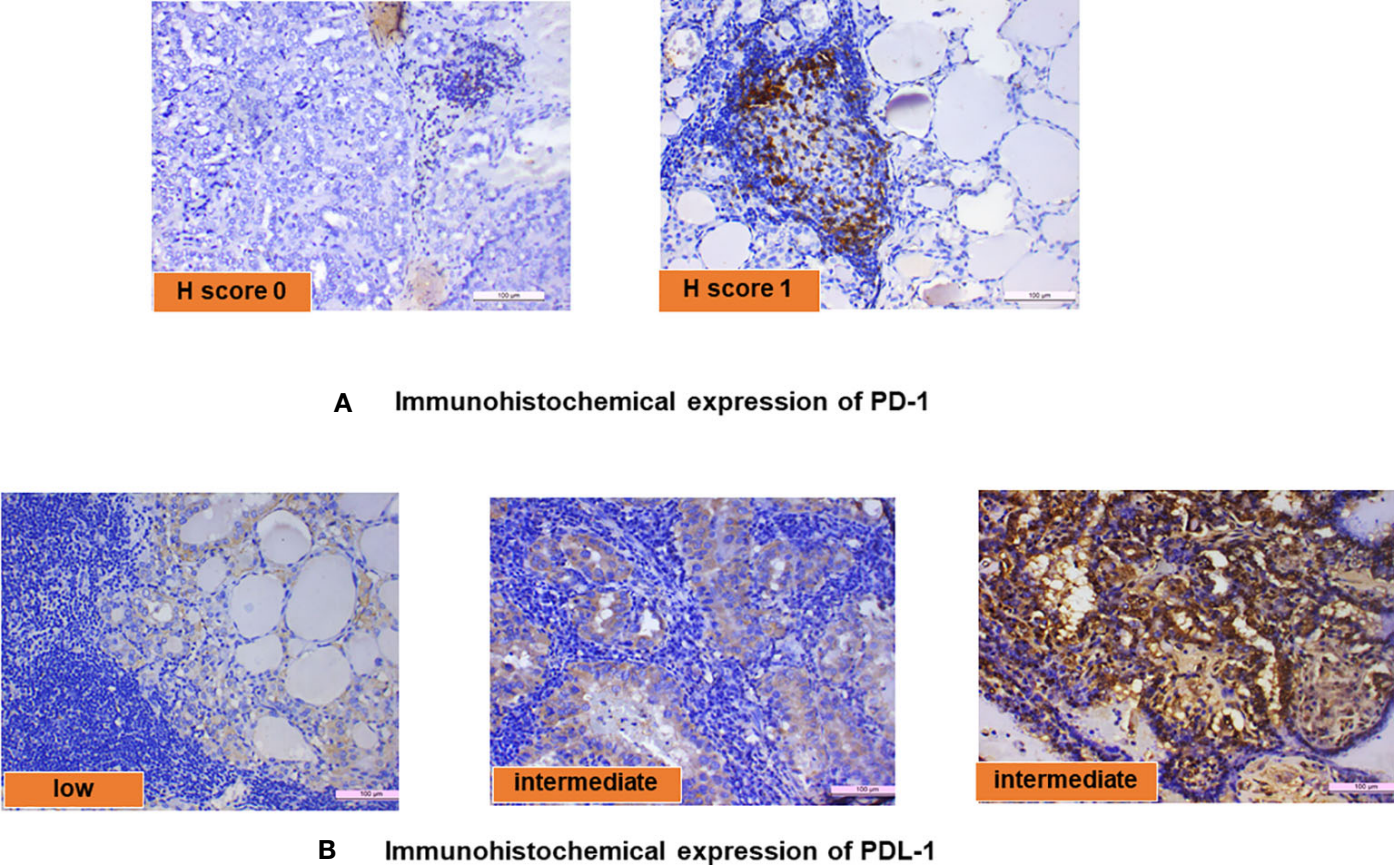
**(A)** Immunohistochemical expression of PD-1 and **(B)** immunohistochemical expression of PD-L1.

The PD-1 and PD-L1 IHC was done on 23 samples of PTC with LT. Of this, PD-1 positivity was noted in 13/23 (56.5%) cases with an intensity varying from 1+ to 2+ (**Figure 3A**). Similarly, PD-L1 positivity was intermediate (1%–49%) in 16/23 (70%) cases and low (<1%) in 7/23 (30%) cases according to TPS scoring (**Figure 3B**). The distribution of average scores for PD-1 and PD-L1 in PTC with LT cases is also depicted in **Figure 2C**.

### Expression of PD-1 and PD-L1 mRNA in PTC with LT

**Figure 4:**
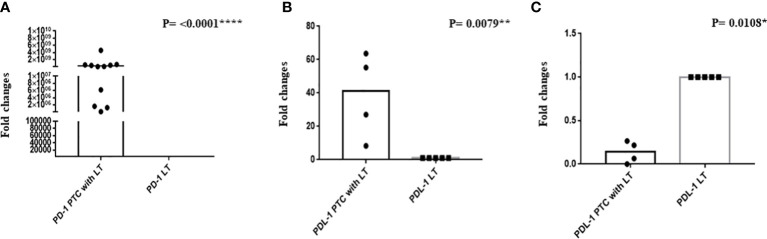
**(A)** Relative mRNA expression of PD-1 and **(B, C)** relative mRNA expression of PD-L1.

qRT-PCR of PD-1 was done in 11 PTC with LT and 7 hyperplastic nodules with LT cases. We observed a significant upregulation of PD-1 mRNA in PTC with LT cases compared with hyperplastic nodules with LT cases (**Figure 4A**, *P* = <0.0001****). PD-L1 expression was significantly upregulated (*P* = 0.0079**) in 50% of the cases, while it was significantly downregulated (*P* = 0.0180*) in 50% of the cases of PTC with LT. However, PD-L1 mRNA downregulation was correlated with negative PD-L1 protein expression (**Figures 4B, C**).

### Clinicopathological association of PD-1 and PD-L1 expression

PD-1 expression was significantly correlated with female gender (**Supplementary Figure 1**), while PD-L1 expression was significantly correlated with the presence of lymph node metastasis (LNM) and the presence of Treg cells in the TME (**Supplementary Figure 1**). The data are depicted in **Table 2**.

**Table 2:** Clinicopathological association of PD-1 and PD-L1 expression.

Parameters	PD-1+	PD-1−	*P*-value
Female, 13/20	65	35	<0.0001
Male, 0/3	0	100	
Parameters	PD-L1+	PD-L1−	*P*-value
LNM+, 11/16	69	31	<0.0001
LNM−, 2/6	33	67	
FoxP3+, 10/12	83	17	<0.0001
FoxP3−, 4/11	36	64	

### Classification of TME based of PD-1 and PD-L1 expression

Based on PD-L1 and PD-1 expression, the TME of PTC with LT cases was subclassified into four categories. In our cohort, 35% of the cases belong to type 1 TME (PD-L1-positive/PD-1-positive), 13% belong to type 2 (PD-L1-negative/PD-1 negative), 26% of the cases were of type 3 TME (PD-L1-positive/PD-1-negative), and 26% of the cases belong to type 4 TME (PD-L1-negative/PD-1-positive).

### Immunophenotyping of TILs from thyroid aspirates by flow cytometry

**Figure 5:**
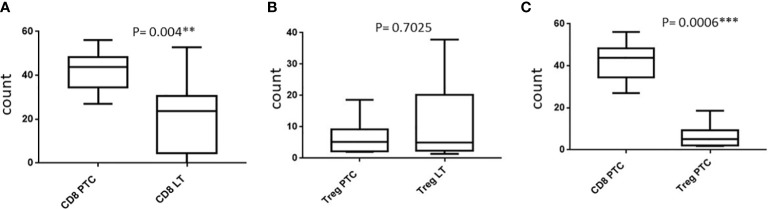
**(A–C)** Relative protein expression in flow cytometry.

We included 7 patients of PTC and 14 patients of LT for FCM of FNAC samples. There was an abundance of CD3^+^CD8^+^ CTLs as compared with CD4^+^CD25^+^ Treg lymphocytes in PTC samples (mean ± SD: 41.31% ± 9.77% *vs*. 27.1% ± 4.5% of the total TIL, respectively; *P* = 0.0006). Furthermore, the FNAC samples from LT showed significantly lower CD3^+^CD8^+^ CTL count accounting for 20.59% ± 16.25% of the total lymphocytic infiltrate as compared with PTC (*P* = 0.004). We observed a lower proportion of CD4^+^CD25^+^ Treg in PTC compared with LT although it was statistically insignificant (*P* = 0.7025). The isolation and relative expression of CD8^+^ CTL and Treg cells by flow cytometry is shown in **Figure 5** and **Supplementary Figure 2**, respectively.

## Discussion

The immunophenotypic characteristic of TILs in PTC and their role in pathogenesis or progression of PTC are not clearly understood. Although the expression of PD-1 receptor on TILs and its ligand PD-L1 in non-thyroidal tumors has been explored, the literature available on the role of the TME in PTC is scarce. We characterized TILs in PTC and analyzed the expression pattern of PD-1 and PD-L1 in the TME.

IHC on FFPE tissues of PTC with LT showed relative abundance of CD8^+^ CTL as compared with FoxP3^+^ Treg cells (83% *vs*. 52%). Moreover, the IHC expression of CD8^+^ CTLs in PTC with LT was comparable to that in hyperplastic nodules with LT as the median *H* score was similar between the groups. Our observation was in concordance with Cunha et al. as they had reported 68% of the cases of PTC with CD8^+^ T-cell infiltration. However, they did not compare cases with hyperplastic nodules with LT cases (19). Modi et al. reported CD8^+^ CTL infiltration in 38% of the cases of PTC which was much lower than our study. They suggested an involvement of cell-mediated immune response in PTC (20). Similar to our findings, Sulaieva et al. also observed that the presence of coexisting LT might lead to increased number of CD8^+^ CTL infiltration in PTC and surrounding thyroid tissue (21). However, Yang et al. observed that CD8^+^ T cells were decreased in advanced stages and with distant metastasis rather than in early stages of PTC (CD8 less in the late stage). In our study, the FoxP3^+^ Treg cells were present in almost similar proportion of PTC with LT (52%) and hyperplastic nodule with LT (53%) cases with a comparable median *H* score. In contrast to our observation, Yu et al. reported higher numbers of FoxP3^+^ Tregs in PTC than in nodular goiter; however, they did not subclassify cases into PTC with and without LT (22). Cunha et al. also observed 91.9% FoxP3 positivity in PTC cases without specifying about the coexistence of LT (23). Similarly, Gogali et al. reported a higher proportion of FoxP3^+^ Treg cells in patients with PTC than in patients with nodular goiter (24). Also, Liu et al. found that the percentage of Treg was increased in peripheral blood as well as in tumor and metastatic lymph nodes of patients with PTC, and there was an absence of FoxP3 expression in multinodular goiter (MNG). However, the same study reported a much lower percentage of Treg cells in peripheral blood and increased infiltration of FoxP3^+^ Treg cells in tumors of PTC with HT patients (25). Their data suggested that the malignant microenvironment may activate Treg infiltration in PTC. However, in IHC, we observed a relative abundance of CD8^+^ CTLs compared with FoxP3^+^ Treg cells in PTC with LT, but the difference was statistically insignificant. This may be due to differences in the TME of our PTC cases because of ethnic variation including variable antigenic expression or their interaction with infiltrating immunological cells.

The function of Treg cells is to suppress CD8^+^ CTLs (26), and the number of CD8^+^ cells reflects the activation of immunologic surveillance, immune elimination, and antitumor response. This response is dependent on the cascade of events including tumor antigen recognition and CD8^+^ cell differentiation with further recruitment of CD8^+^ T cells into the tumor site followed by tumor cell elimination. Pilli et al. described that the CD8^+^/FoxP3^+^ T lymphocyte ratio may represent the balance between the immune attack against cancer cells and immune tolerance. They reported a lower immune tolerance at the peritumoral area in more aggressive thyroid cancer patients who were not cured. Their observation suggested that a higher number of regulatory T cells resulted in lesser tumor destruction by the immune system (27). On the contrary, Cunha et al. reported a higher number of Tregs in smaller tumors and those without extrathyroidal invasion. However, all the samples in their cohort had concurrent lymphocytic thyroiditis (28).

Issa-Nummer et al. described that a high number of cytotoxic T cells contributed to antitumor effects in breast cancer and other malignancies (29). Furthermore, French et al. confirmed the association of a higher number Treg cells with invasiveness and aggressiveness of PTC by exerting an immunosuppressive effect in the TME (30). Additionally, Zou et al. observed that a high number of Treg cells facilitated tumor progression through production of numerous proangiogenic factors and suppression of tumor immune response (31).

We observed predominant CTL infiltration as compared with Tregs in the TME of PTC with LT. Similar to the PTC cases in our study, immunophenotyping of infiltrating lymphocytes in thyroid tissue of HT showed the predominance of CD8^+^ T cells. This resulted in thyrocyte destruction due to an altered CD8^+^ CTL and Treg cell ratio through an apoptotic process leading to hypothyroidism (32). However, we noted that despite the abundance of CD8^+^ CTL in the TME of PTC, there was the absence of clinical or subclinical hypothyroidism due to thyrocyte destruction or tumor cell elimination, unlike HT. Hence, additional mechanisms should play a role which enables the PTCs to survive and grow in a hostile microenvironment, either by exhaustion or inactivation of infiltrating cytotoxic T lymphocytes. It was described by Farhood et al. that tumor growth in the presence of CD8^+^ cell infiltration may be associated with abnormalities of tumor cell recognition and killing related to CD8^+^ T-cell disability or immune escape mechanism activation (33).

Similarly, on flow cytometry of FNAC samples, we found an abundance of CD8^+^ CTLs in PTC than in LT. Additionally, in the same samples of PTC, CD8^+^ CTLs were more predominantly observed than FoxP3^+^ Tregs. Immunophenotyping of FNAC samples showed higher CD8^+^ CTL count and statistically insignificant but lower CD4^+^CD25^+^ Treg count in PTC than in LT. Furthermore, there was a significant abundance of CD8^+^ CTL compared with CD4^+^CD25^+^ Treg lymphocytes in the same samples of PTC. Zhu et al. also observed significantly higher CD8^+^ T cells in the peripheral blood and tumor of PTC with HT patients compared with healthy controls (34). A similar study comparing the immunophenotypic characteristics of infiltrating lymphocytes in FNAC samples of PTC *vs*. benign LT is scarce in the literature. In contrast to our findings, Gogali et al. observed increased Treg but similar CD8^+^ CTL infiltration in PTC cases than in cases with nodular goiter in flow cytometric analysis of tissue sample suspension (24). The predominant antitumor immune response rather than the immune-suppressed status in the TME of PTC in our study may suggest a variation in tumor antigen expression and their interaction with the host immune system.

On further analyzing PD-1 and PD-L1 expression in FFPE tissues of PTC with LT by IHC, we observed positive PD-1 expression in 13/23 (56.5%) cases and intermediate PD-L1 expression on tumor cell membrane in 16/23 (70%) cases. The PD-1–PD-L1 pathway has a vital role in tumor progression and survival as it enables to escape tumor-neutralizing immune response generated in the TME. The interaction of PD-1 receptor on CD8^+^ T cells and its ligand PD-L1 on tumor cells mediates tumor progression and helps the tumor to escape immune surveillance as described in malignancies including melanoma, non-small cell lung carcinoma, colorectal cancer, and renal cell carcinoma (35–37).

The PD-1 receptor is a 50–55-kDa transmembrane glycoprotein present in TILs (38). Various cell membranes, including tumor cells, express its ligands which are PD-L1 and PD-L2, and the latter may be less prevalent (39, 40). The PD-L1 is aberrantly expressed on tumor cells after stimulated by oncogenic signaling pathways (41). Also, proinflammatory cytokines like IFN-γ and IL-2 secreted by activated T cells in the TME can stimulate its expression (42). Previously, Zhu et al. confirmed PD-1 expression on CD8^+^ T cells in the peripheral blood and tumor of PTC with HT patients. They also reported a significantly higher expression of PD-L1 in tumors of PTC with HT patients than in patients with nodular goiter (34).

To validate the results, quantitative real-time PCR was done which revealed significant upregulation (*P* < 0.05) of PD-1 mRNA in PTC with LT. However, significant upregulation (*P* < 0.05) of PD-L1 mRNA was seen in 50% of these cases compared with hyperplastic nodules with LT cases. Similarly, Bastman et al. reported elevated PD-1 mRNA expression in CD4^+^ and CD8^+^ T cells infiltrating differentiated thyroid carcinoma which could be due to exposure to tumor antigens (43). Furthermore, PD-1–PD-L1 interaction may cause dephosphorylation of cytoplasmic Src homology 2 (SH2) domain containing phosphatase 2 (SHP2) on the intracellular segment of PD-1. This results in immune tolerance *via* suppression of cytotoxic T-cell-mediated immune response (CD8^+^ T-cell exhaustion) (44). This is followed by interleukin-10 (IL-10) production in tumor mass (45) with consequent tumor progression by inhibiting effector T-cell function in the TME (46). Thus, targeting PD-1 or PD-L1 can revert the function of effector T cells, thereby resulting in the elimination of cancer cells. In this context, immune checkpoint inhibitors which block PD-1 and PD-L1 interaction have been used in various advanced malignancies and metastatic disease for better therapeutic effect (47–49).

We found a significant association between PD-1/PD-L1 expression and clinicopathological parameters like female gender, presence of LNM, and Treg cell expression in our study. PD-L1 expression in association with LNM and Treg cell expression may correlate with the aggressive behavior of PTC. In contrast with our findings, Bai et al. could not find any correlation of PD-1+ T-cell frequency with aggressive clinicopathological features including a large tumor size and lymph node metastases (16). However, in another study by the same authors, a significant correlation between PD-L1 expression and female gender was observed (13). In other cancers like cutaneous squamous cell carcinoma, a high-intensity score of PD-L1 rather than proportion was directly correlated with LNM (50). Similarly, Alves et al. observed more PD-L1 expression in lymph node metastasis rather than in primary tumors of breast cancer; however, they could not find its association with T-cell exhaustion markers (51). Additionally, Uruga et al. observed a higher rate of PD-L1 expression in the primary tumor associated with nodal metastasis and in stage II and III lung adenocarcinoma with adverse pathologic features. They confirmed that abundant CD8^+^ tumor-infiltrating T cells were the only predictor of PD-L1 expression in their study (52). Another study has demonstrated more frequent and stronger PD-L1 expression in LNM rather than in primary tumor cells of triple-negative breast cancer (TNBC). These observations may increase the accuracy of predicting patients’ prognosis which may further allow better treatment selection (53).

In our study, the expression pattern of PD-1 and PD-L1 at the protein and mRNA levels suggested that PTCs might be evading immune surveillance by exhausting CD8^+^ CTLs through PD-1 and PD-L1 interaction. Thus, targeting PD-1 and PD-L1 interaction (blocking) can revert back the function of effector T cells. This could further be utilized in radioactive iodine-resistant PTC cases, where immune checkpoint inhibitors which can block PD-1/PD-L1 interaction may be useful as a novel therapeutic approach. Although data on thyroid cancer are limited, immune checkpoint inhibitors have shown to be effective in various other cancers even in advanced stage and may be a potential therapeutic option for thyroid cancer cases as well. Furthermore, a phase II trial of pembrolizumab (a PD-1 inhibitor) is being conducted in metastatic or locally advanced anaplastic/undifferentiated thyroid cancer (clinicaltrial.gov.in NCT05119296). Another phase II clinical trial of nivolumab (a PD-1 inhibitor) is being conducted in metastatic radioiodine-resistant BRAF V600E-positive thyroid cancer (clinicaltrial.gov.in NCT04061980). Also, durvalumab (a PD-L1 inhibitor) is being used in a phase II clinical trial of progressive, refractory advanced thyroid carcinoma (clinicaltrial.gov.in NCT03753919). However, more phase III clinical studies are needed in radioiodine-resistant PTC cases. Furthermore, we found that PD-L1 expression was associated with lymph node metastasis and Treg cell infiltration in PTC. Thus, PD-L1 positivity is related with aggressive behavior of the tumor and adverse prognosis and may require more intensive follow-up by the clinician.

There are some limitations in our study. A total of 29 PTC patients were recruited in this study, of which IHC was done in 23 patients and FCM was done in only 7 patients. It would be better if we could have done IHC as well as FCM in all the patients. However, due to limited availability of resources and funding, we had to restrict the wet lab experiments. Still, we analyzed the results separately with their respective controls. The second limitation of the study is that although we have incorporated information on overall survival, we have data regarding progression-free survival (PFS) of only 69% (20/29) of the patients due to the COVID-19 pandemic and irregular follow-up. Third, we have a relatively small sample size. A larger cohort study in this scenario would be helpful in getting more significant results. We wanted to expand the study period to collect more samples; however, due to the COVID-19 pandemic and administrative lockdown, we had to stop further patient recruitment.

## Conclusion

The characterization of the TME in PTC with LT may open up new paradigms for targeted therapy in these patients. We found the existence of antitumor immunity in PTC with LT cases, with relative abundance of CD8^+^ CTL in the TME which was not associated with tumor cell elimination. The CD8^+^ CTL in the TME might be rendered ineffective by the expression of its co-inhibitory receptor PD-1, which is also a receptor for PD-L1 expressed by the malignant cells. PD-L1 expression was significantly associated with the aggressive behavior of PTC. Thus, PD-1/PD-L1 blockade may prove to be a useful immunotherapeutic approach in patients of PTC with LT, particularly in metastatic radioiodine-resistant cases, which require further studies targeting this subgroup of PTC cases.

## Data availability statement

The raw data supporting the conclusions of this article will be made available by the authors, without undue reservation.

## Ethics statement

The studies involving human participants were reviewed and approved by Institute Ethics committee (IEC), PGIMER Chandigarh. The patients/participants provided their written informed consent to participate in this study.

## Author contributions

SoB conceptualized the study design, conducted all the experiments included, reviewed the literature, and wrote the manuscript. UN conceptualized the study design, reviewed the literature, and edited the manuscript. DD recruited the patients and provided the clinical details, provided the tissue samples, and edited the manuscript. SM recruited the patients and provided the clinical details, reviewed the literature, and edited the manuscript. PD provided the FNAC samples, conceptualized the study design, and edited the manuscript. RG recruited the patients and provided the clinical details, provided the tissue samples, and edited the manuscript. BR and NS conceptualized the study design, reviewed the literature, and edited the manuscript. AS recruited the patients and provided the clinical details, reviewed the literature, and edited the manuscript. SaB and AB conceptualized the study design, recruited the patients and provided the clinical details, and edited the manuscript. All authors contributed to the article and approved the submitted version.

## Funding

The study was supported by grants (71/2-Edu-16/119) through the Intramural Research Committee of the Postgraduate Institute of Medical Education and Research, Chandigarh.

## Acknowledgments

We would like to acknowledge the Postgraduate Institute of Medical Education and Research (PGIMER), Chandigarh for allowing us to conduct this study.

## Conflict of interest

The authors declare that the research was conducted in the absence of any commercial or financial relationships that could be construed as a potential conflict of interest.

## Publisher’s note

All claims expressed in this article are solely those of the authors and do not necessarily represent those of their affiliated organizations, or those of the publisher, the editors and the reviewers. Any product that may be evaluated in this article, or claim that may be made by its manufacturer, is not guaranteed or endorsed by the publisher.
