# Supplementary figures and images for: Role of cytotoxic T cells and PD-1 immune checkpoint pathway in papillary thyroid carcinoma
Source: Front Endocrinol (Lausanne). 2022 Nov 28;13:931647. doi: 10.3389/fendo.2022.931647 (PMC9742369; doi:10.3389/fendo.2022.931647)

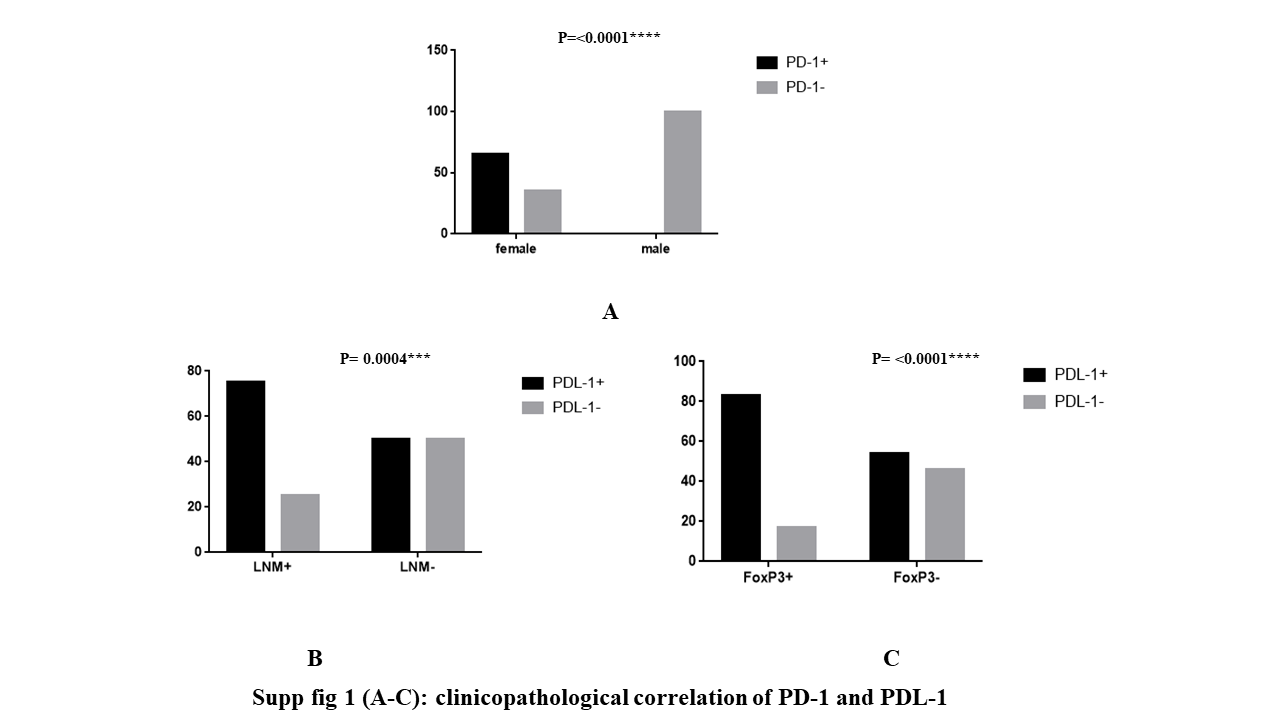

Supplement: Supplementary file 1 [file Image_1.tif]

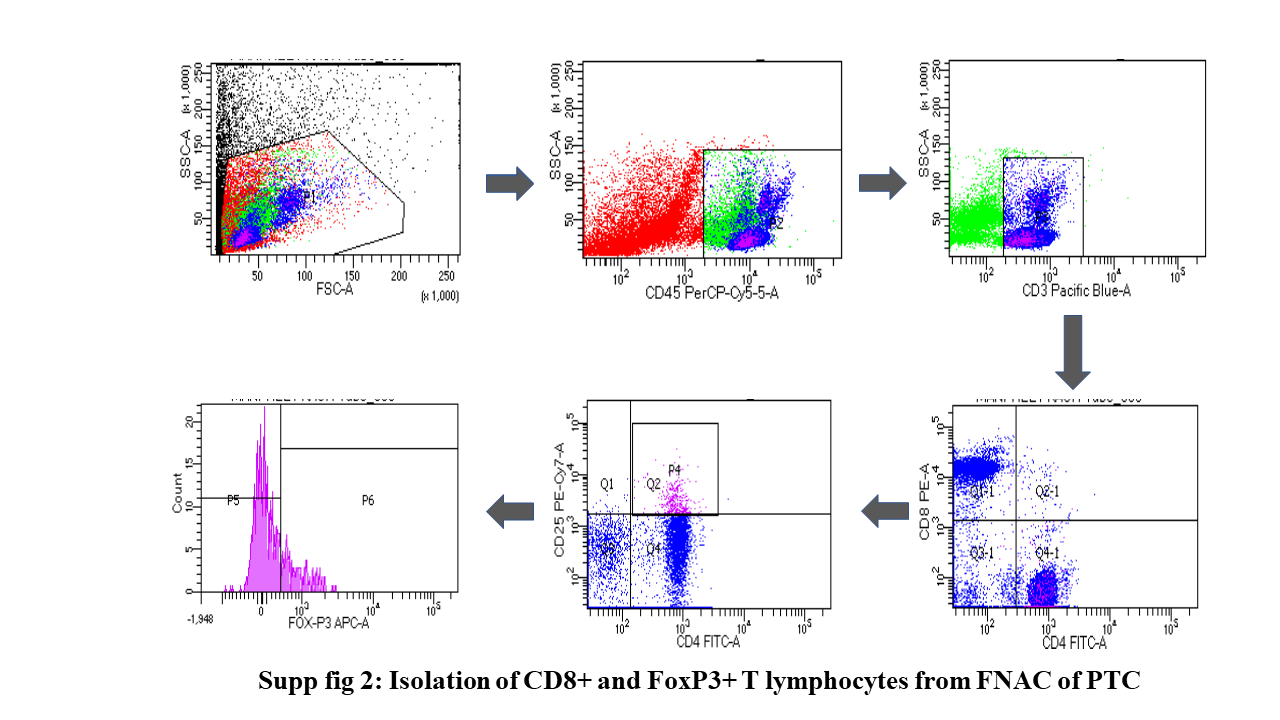

Supplement: Supplementary file 2 [file Image_2.tif]
